# On the Pathology of Hooping-Cough

**Published:** 1831-01-01

**Authors:** 


### ON THE PATHOLOGY OF HOOPING-COUGH. By *James Alderson*, M.D. Physician to the Hull General Infirmary.

[Med. Chir. Trans. Vol. XVI.]

DR. A. considering it strange that the pathology of this disease should be so much neglected, and that its treatment should be left so much to nurses and nostrum-mongers, has gladly availed himself of numerous opportunities of post-mortem examinations, in order to ascertain whether the general opinion of physicians respecting the non-inflammatory nature of simple pertussis be correct. He observes that when the physician is called in, and the case proves fatal, death is not ascribed to hooping-cough, but to inflammation of the lungs, or convulsions, "the most prominent symptom of the fatal stage of the disease." As the appearances on dissection, in all the cases which our author examined, were precisely the same, he conceives we are justified in considering them as "the effects of the continued and uncontrolled action of hooping-cough." We need not follow Dr. A. in his observations on the structure of the lungs, or his description of the symptoms of hooping-cough; but proceed to the cases and dissections—the most important portions of the paper.

"*Case* 1. On Monday, 26th March, 1828, I visited Sarah Allison, aged two years, residing in Blewett's Building's, Fetter Lane; she lay upon the left side; respiration difficult, irregular, and nearly the whole act taking place by the diaphragm alone; pulse very frequent; face puffy and livid; lips purple; nostrils dry; alæ of the nose in considerable action; tongue white; countenance anxious; skin of the body hot; great thirst; extremities cold; frequent paroxysms of cough, during which the child put its little fingers down its throat to get rid of the phlegm; bowels open. She had been for three weeks affected with Hooping Cough, and several children had died of the same disease in the neighbourhood. She was freely leeched and blistered, and took saline mixture with digitalis and conium, with warm bath to the lower extremities; she took no other food than barley-water.

On the 29th, difficulty of breathing increased; great restlessness; face livid; respiration very irregular and abdominal; flatulent distension of the abdomen. In the evening, the hands became clenched and turned inwards; face puffy; jaw locked; each side of the body in turns convulsed, and she died in a few hours.

*Dissection*, thirty-six hours after death.—There were no adhesions of the pleura, nor any fluid in the cavity of the chest; the lungs did not collapse, they contained much air, which was with difficulty pressed out: in different parts of the lungs, but chiefly in the lower portions of all the lobes, a change of structure had taken place, which has been termed lobular hepatization; this was more evident in the middle lobe of the right lung, which was quite contracted to the size of the pancreas, and which it very much resembled in the sensation which it conveyed to the touch; the septa dividing the lobules were distinctly seen, and appeared perfectly healthy, the lobules themselves quite dense, and somewhat resembling the muscular structure of the heart; several of the smaller bronchial tubes were much dilated, and lined with thick mucus; there was no appearance of inflammation in the trachæa or in the larger bronchial tubes.

*Case* 2. John Allison, aged 7½ months, brother to the preceding; a remarkably strong and vigorous child, had been affected for a fortnight with Hooping Cough; but as he took the breast freely, and was very lively, little had been thought of his complaints. I found him, on the 11th of April, 1828, breathing with great difficulty; respirations 62 in a minute, irregular and abdominal; nostrils dry, and alæ of the nose in great action; pulse 140; tongue white and furred. Three leeches were applied to the chest, and a small blister, for four hours, between the shoulders; a grain of calomel and one-eighth of a grain of tartarized antimony, every eight hours. On the 13th he had several convulsion fits, and the abdomen became distended with flatus. The evacuations, like chopped grass and offensive, passed during the paroxysms of cough. 14th. Very sick from the antimony; had taken the breast several times, but as often vomited the milk. Bowels freely purged; motions not so green and offensive. 15th. System affected by mercury; tongue white, dry, and swollen; inner parts of the mouth and lips blistered, nostrils dry, skin of the upper parts of the body intensely hot, extremities cold, face flushed, lips purplish, breathing more laborious, catching, and abdominal. Died in the afternoon.

*Dissection*, forty hours after death, carefully made for me by my talented friend Dr. Hodgkin. The head was rather large, the forehead remarkably protuberant; the calvaria was not raised with greater difficulty than usually attends the operation in the infant. The quantity of serum in the ventricles and under the arachnoid coat was rather larger than was natural. The substance of the brain was of the soft consistence natural to the organ at the age of the subject. The only morbid appearance observable except the before-mentioned slight effusion, was an irregular venous marbling of the medullary matter. There was no trace of pleuritic inflammation on either side of the chest. About one-fourth of both lungs, towards their posterior and inferior part, was the subject of a morbid change; the structure was rendered very firm and dense; its limits were perfectly defined, the septa between the lobules constituting the boundaries. The portions in which this change had taken place were of a dull red colour, perfectly void of air, sinking instantly when put into water, and undergoing no change when thin slices were subjected to ablution in it." 85.

Dr. A. informs us that, in many other cases which he examined, he found the very same appearances, "uncomplicated with any evidence of pleuritic inflammation." In the lower and posterior portions of the lungs, the structure was rendered very firm and dense—the portions which were the subject of this change being exactly defined by the septa—of a dull red colour—devoid of air—sinking instantly in water—and thin slices undergoing no change by ablution. The individual lobules, he observes, were more dense than in hepatized lungs, and the cellular membrane between them retained its natural structure, conveying to the touch the same sensation that is felt on handling the pancreas. The upper portions of lung were spongy and crepitant; but little air could be expelled from them by the bronchial tubes. Most of the smaller tubes were filled with a thick secretion—in one case with a false membrane—in all, the divisions of the tubes were somewhat dilated.

Dr. A. remarks that—"in the treatment of this stage of the complaint, no time should be lost in the application of remedies; the approach to a fatal termination is rapid, and nearly the same promptitude is necessary here as in croup. Leeches are too slow in their mode of extracting blood, to be of effectual service." He has, however, been much satisfied with the result of the prompt and bold extraction of blood by cupping, followed by calomel and antimony, or James's powder in small doses, repeated at short intervals, until the symptoms above detailed give way to others less distressing. Secretion of mucus then takes place, he thinks, and the adhesive membrane is absorbed or thrown off by expectoration—the breathing becomes less laborious—and the lips assume a more natural hue. More blood, he observes may be taken in these cases, than we would be led to consider requisite or safe under other circumstances.

We do not deem it necessary to make many remarks on the above paper. We believe, from some observation, that the foregoing appearances of hepatization will be generally found in the lungs of children who have been carried off by violent hooping-cough. And however we may dispute about the terms inflammation, congestion, or hepatization, the practical conclusions to which we must come at last are these—that, in some cases of hooping-cough, there is an accompanying or supervening condition of the lungs, which adds greatly to the danger and difficulty of the case, and requires prompt and decisive depletion, to prevent death or serious change of structure in the respiratory apparatus.

We wonder Dr. A. has not alluded to the state of the head in these violent cases of hooping-cough—and that he has no where given any examinations of the brain. Without going so far as our ingenious and talented friend, Dr. Webster has gone, in placing the cause of pertussis in the head, we can safely say that we have witnessed very few fatal instances of the disease, where the symptoms as well as the dissections did not shew serious affection of the brain. Indeed we are greatly inclined to attribute the immediate cause of death more to this secondary than to the primary malady. Moreover, we strongly recommend the practitioner to bear this in mind at the bed-side of the little patient; and to direct his attention to the relief of the head as well as of the chest, by leeches to the temples and behind the ears. We have recently seen three cases, which appeared almost hopeless, rescued apparently from the jaws of death, by repeated leechings of the head. We beg to return our thanks, however, to Dr. Alderson for his communication on this important subject.

